# Supplementary material for: A Contemporary Review of Antiplatelet Therapies in Current Clinical Practice
Source: Int J Mol Sci. 2023 Jul 5;24(13):11132. doi: 10.3390/ijms241311132 (PMC10342577; doi:10.3390/ijms241311132)
Supplement: Supplementary file 1 [file ijms-24-11132-s001.zip › ijms-2371799-supplementary.pdf]

Online appendix – Tables of notable trials included in the review

Legend:

Table S1: Randomised studies of antiplatelet therapy in primary prevention for ASCVD

Table S2: Randomised studies of antiplatelet therapy after elective PCI for CCS

Table S3: The main randomised studies of antiplatelet therapy after ACS

Table S4: The main randomised studies of antiplatelet treatment strategies after CABG

Table S5: Long-term antiplatelet therapy for secondary prevention in IHD patients

Table S6: Randomised studies of antiplatelet treatment strategies after TAVI

Table S7: Randomised studies of antiplatelet therapy in stroke and TIAs

Table S8: Randomised studies of antiplatelet therapy in peripheral artery disease

Table S9: Randomised studies of antiplatelet treatment strategies for COVID patients

Table S1: Randomised studies of antiplatelet therapy in primary prevention for ASCVD

Trial	Participants	Setting	Treatment	Primary endpoint	Main findings
**ASCEND** [1]<br>**2018**	N = 15,480.<br>Mean age 63 years.	Randomised clinical trial.<br>Evaluating aspirin vs. placebo among diabetics with no known cardiovascular disease.<br>Follow up: 7.4 years.	Aspirin 100 mg daily vs. placebo.	MACE; vascular death, MI, or stroke/TIA.<br>Primary safety outcome of major bleeding.	The absolute reduction in primary cardiovascular events from aspirin was offset by a similar absolute increase in major bleeding.<br>MACE occurred in 8.5% of the aspirin group vs. 9.6% of the placebo group (p = 0.01).<br>Major bleeding occurred in 4.1% of the aspirin group vs. 3.2% of the placebo group (p = 0.003).
**ASPREE** [2]<br>**2018**	N = 19,114.<br>Median age 74 years.	Randomised clinical trial.<br>Low-dose aspirin vs. placebo among healthy elderly patients.<br>Median follow up: 4.7 years.	Aspirin 100 mg daily vs. placebo.	All-cause death, dementia, or physical disability	Aspirin did not prevent disability-free survival, but did increase major bleeding compared with placebo.<br>Primary outcome: 21.5 events per 1,000 person-years in the aspirin group vs. 21.2 events per 1,000 person-years in the placebo group (p = 0.79).
**ARRIVE** [1]<br>**2018**	N = 12,546.<br>Mean age 64 years.	Randomised clinical trial.<br>Aspirin vs. placebo among patients with moderate risk of cardiovascular	Aspirin 100 mg daily vs. placebo.	Primary efficacy outcome: Cardiovascular death, MI, unstable angina, stroke, or TIA	In patients at moderate risk of cardiovascular disease, aspirin was not beneficial. Aspirin was not associated with a reduction in primary adverse cardiovascular events (4.3% of the aspirin group vs. 4.5% of the placebo group; p = 0.60).

		disease (10-year risk of coronary heart disease 10-20%). Non-diabetics.<br><br>Median follow up: 60 months.		Primary safety outcome: gastrointestinal bleeding	Bleeding rates were low and similar overall (0.97% in the aspirin group vs. 0.46% in the placebo group; p = 0.0007).
**PolyIran** [3]<br><br>**2019**	N = 1,508.<br><br>Mean age 58.6 years in the polypill arm; 59.4 years in the control arm.	Randomised clinical trial.<br><br>Assessing a fixed-dose polypill containing aspirin, atorvastatin, hydrochlorothiazide, and valsartan on primary prevention of major cardiovascular events.<br><br>Follow up: 60 months.	Polypill contained 81 mg aspirin, 12.5 mg hydrochlorothiazide, 20 mg atorvastatin, and 40 mg valsartan.	MACE, including fatal MI, sudden death, new-onset heart failure, coronary artery revascularisation procedures, fatal and non-fatal stroke, or hospitalisation for an acute coronary event.	The polypill resulted in a reduction in the incidence of MACE (8.0% polypill vs. 11.9% control, p = 0.002).

Abbreviations: CI = Confidence Interval; HR = Hazard Ratio; MACE = Major Adverse Cardiovascular Events; MI = Myocardial Infarction; NSTEMI = Non-ST Elevation Myocardial Infarction; TIA = Transient Ischaemic Attack.

Table S2: Randomised studies of antiplatelet therapy after elective PCI for CCS

Trial	Participants	Setting	Treatment	Primary endpoint	Main findings
**CREDO[4]**<br>**2002**	N=2116 patients undergoing elective PCI stable angina, unstable angina, or recent MI (indication for PCI stable angina in 33%)	Randomised controlled trial<br><br>Double-blind	300mg clopidogrel 3 to 24 hours before PCI, then DAPT with aspirin plus clopidogrel for 12 months vs. placebo pre-PCI, then DAPT with aspirin plus clopidogrel for 28 days, followed by placebo and aspirin until 12 months.	Composite of death, MI, or stroke, at 1 year.	Lower risk of the primary endpoint after 1 year DAPT with aspirin plus clopidogrel vs. 28 days DAPT (26.9% relative reduction [95% CI: 3.9% to 44.4%] p=0.02).<br><br>Subgroup analysis suggests that longer intervals between clopidogrel loading dose and PCI may reduce the composite of death, MI and urgent target vessel revascularisation (clopidogrel loading ≥ 6 hours pre-PCI, (38.6% relative reduction [95% CI: -1.6% to 62.9%] p=0.051).
**EXCELLENT[5]**<br>**2012**	N=1443 patients (indication for PCI stable angina in 48%)	Randomised controlled trial<br><br>Open label	6 months of DAPT (aspirin plus clopidogrel) thereafter aspirin alone vs. 12 months DAPT	Target vessel failure defined as the composite of cardiac death, MI, or ischaemia-driven target vessel revascularization at 1 year	Six month DAPT did not increase of the primary endpoint vs. 12 months DAPT 4.8% vs. 4.4%, p=0.001 for non inferiority).
**SECURITY[6]**<br>**2014**	1399 patients with stable or unstable angina (indication for PCI	Randomised non-inferiority trial	6 months of DAPT (aspirin plus clopidogrel)	Composite of cardiac death, MI, stroke, definite or probable stent thrombosis, or major or clinically	Six months DAPT was non-inferior to 12 months DAPT with respect to the primary endpoint (4.5% vs. 3.7%; risk

	stable angina in 38%)		thereafter aspirin alone vs. 12 months DAPT	relevant non major bleeding at 1 year	difference: 0.8% [95% CI: -2.4% to 1.7%], p=0.469).
**ISAR-SAFE[7]**<br><br>**2015**	N=4000 patients on DAPT (aspirin and clopidogrel) 6 months after stent implantation (indication for PCI stable angina in 48%)	Randomised controlled trial<br><br>Double-blind	After 6 months of DAPT (aspirin plus clopidogrel) randomised to 6 months of aspirin vs. placebo vs. 6 months of DAPT (aspirin plus clopidogrel)	Composite of death, MI, stent thrombosis, stroke and major bleeding at 9 months after randomisation	Six months DAPT was non-inferior to 12 months DAPT with respect to the primary endpoint (1.5% vs. 1.6%, p<0.001 for non inferiority).
**GLOBAL LEADERS[8]**<br><br>**2018**	N=15968 patients undergoing PCI for stable coronary disease or ACS (indication for PCI stable coronary disease in 53%)	Randomised superiority trial<br><br>Open label	Experimental arm: DAPT with aspirin plus ticagrelor for 1 month, then 23 months of ticagrelor monotherapy vs. control arm: DAPT with aspirin plus either clopidogrel or ticagrelor for 12 months, followed by SAPT with aspirin	Composite of all-cause mortality, or non-fatal new Q wave MI at 2 years	No difference in primary endpoint between experimental arm vs. control arm (3.8% vs. 4.4%; rate ratio: 0.87 [95% CI: 0.75 to 1.01] P=0.073).<br><br>No difference in major bleeds between experimental arm vs. control arm (2.0% vs.2.1%; rate ratio: 0.97 [95% CI: 0.78 TO 1.20] p=0.77).
**MASTER DAPT[9]**<br><br>**2021**	N=4434 patients who underwent successful PCI with the Ultimaster stent for acute or chronic coronary	Randomised, non-inferiority trial, with sequential superiority testing	Experimental arm: discontinuation of DAPT (aspirin plus clopidogrel) after 1 month (median DAPT duration 34 days) vs. control arm: continue DAPT for ≥ 2 additional	3 co-primary endpoints:<br><br>Net adverse clinical events (composite of all-cause death, MI, stroke or major bleeds).	One month DAPT noninferior to the continuation of DAPT for ≥2 additional months, for net adverse events (difference in cumulative incidence: -0.23 percentage points [95% CI: -1.80 to 1.33] p<0.001 for noninferiority).

	syndromes (indication for PCI stable angina in 42%)	Open label	months (median DAPT duration 193 days)	Major adverse cardiac or cerebral events (composite of all cause death, MI, or stroke)<br><br>Major or clinically relevant nonmajor bleeding at 1 year	One month DAPT noninferior to the continuation of DAPT for ≥2 additional months, for major adverse cardiac or cerebral events (difference in cumulative incidence: 0.11 percentage points [95% CI: -1.29 to 1.51] p=0.001 for noninferiority).<br><br>One month DAPT resulted in lower incidence of bleeds vs. longer duration DAPT (difference in cumulative incidence: -2.82 percentage points [95% CI: -4.40 to -1.24] p<0.001 for superiority).
**STOPDAPT 2[10]**<br><br>**2019**	N=3009 patients undergoing PCI for acute or chronic coronary syndromes (indication for PCI stable angina in 62%)	Randomised controlled trial<br><br>Open label	Experimental arm: 1 month DAPT (aspirin plus clopidogrel) then clopidogrel monotherapy vs. control arm: 12 months DAPT (aspirin plus clopidogrel)	Composite of cardiovascular death, MI, stroke, stent thrombosis, or bleeding at 1 year	Lower risk of the primary endpoint after 1 month DAPT vs. 12 months DAPT (2.4% vs.3.7%, hazard ratio: 0.64 [95% CI: 0.42 to 0.98])
**One-month DAPT[11]**<br><br>**2021**	N=3020 patients undergoing PCI (indication for PCI	Randomised non-inferiority trial	Experimental arm: 1 month DAPT (aspirin plus clopidogrel) then aspirin monotherapy	Composite of cardiac death, nonfatal MI, target vessel revascularisation, stroke, or major bleeding at 1 year	One month DAPT was non-inferior to 6 to 12 months DAPT for the primary endpoint (5.9% vs. 6.5%; absolute

	stable angina in 61%)	Open label	(median DAPT duration: 1.1 months) vs. control arm: 6 – 12 months DAPT (aspirin plus clopidogrel) (median DAPT duration: 12 months)		difference: -0.7%, p<0.001 for non-inferiority).

Abbreviations: CI = confidence interval; CREDO = Clopidogrel for the Reduction of Events During Observation; DAPT = dual antiplatelet therapy; EXCELLENT = Efficacy of Xience/ Promus Versus Cypher to Reduce Late Loss After Stenting; GLOBAL LEADERS = A Clinical Study Comparing two Forms of Anti-platelet Therapy After Stent Implantation); ISAR-SAFE = Safety and Efficacy of Six Months Dual Antiplatelet Therapy After Drug-Eluting Stenting; MASTER DAPT = Management of High Bleeding Risk Patients Post Bioresorbable Polymer Coated Stent Implantation With and Abbreviated Versus Prolonged DAPT Regimen; MI = myocardial infarction; SAPT = single antiplatelet therapy; SECURITY = Second Generation Drug-Eluting Stent Implantation Followed by Sex- Versus Twelve-Month Dual Antiplatelet Therapy; STOPDAPT-2 = ShorT and Optimal Duration of AntiPlatelet Therapy-2 Study

Table S3: The main randomised studies of antiplatelet therapy after ACS

Trial	Participants	Setting	Treatment	Primary endpoint	Main findings
**CURE[12]**<br><br>**2001**	N=12562 patients with NSTEMI or unstable angina	Randomised controlled trial<br><br>Double-blind	DAPT (aspirin plus clopidogrel) for 3 to 12 months vs. aspirin and placebo	Composite of cardiovascular death, MI or stroke at 1 year	Lower risk of the primary endpoint with DAPT vs. aspirin monotherapy (9.3% vs. 11.4%; relative risk: 0.80 [95% CI: 0.72 to 0.90) p<0.001).<br><br>More major bleeds with DAPT vs. aspirin monotherapy (3.7% vs. 2.7%; relative risk: 1.38, p=0.001).<br><br>No difference in life-threatening bleeds between DAPT vs. aspirin monotherapy (2.1% vs. 1.8%, p=0.13).
**CLARITY-TIMI 28[13]**<br><br>**2005**	N=3491 patients with STEMI treated with fibrinolysis	Randomised controlled trial<br><br>Double-blind	DAPT (aspirin plus clopidogrel) vs. aspirin and placebo until coronary angiography, or if angiography not done, hospital discharge or day 8 of	Composite of an occluded infarct-related artery on angiography, or death or MI before angiography at 30 days	By 30 days clopidogrel reduced the odds of the primary endpoint by 20% (from 14.1 to 11.6%, p=0.03).

			hospital admission.		
**PLATO[14]**<br><br>**2009**	N=18624 patients with ACS, with or without ST-segment elevation	Randomised controlled trial<br><br>Double-blind	DAPT with aspirin plus ticagrelor vs aspirin plus clopidogrel	Composite of death from vascular cause, MI or stroke at 1 year.	Lower risk of the primary endpoint with ticagrelor vs. clopidogrel (9.8% vs. 11.7%; hazard ratio: 0.84 [95% CI: 0.77 to 0.92] p<0.001).<br><br>No difference in the rates of major bleeding between the ticagrelor vs. clopidogrel groups (11.6% vs. 11.2%, p=0.03).
**TRITON-TIMI 38[15]**<br><br>**2007**	N=13608 patients with high-risk ACS scheduled for PCI	Randomised controlled trial	DAPT with prasugrel plus aspirin vs. clopidogrel plus aspirin for 6 to 15 months	Composite of cardiovascular death, MI, or stroke.	Lower rik of the primary endpoint with prasugrel vs. clopidogrel (9.9% vs. 12.1%; hazard ratio: 0.81 [95% CI: 0.73 to 0.90] P<0.001).<br><br>The risk of major bleeding was higher with prasugrel vs. clopidogrel (2.4% vs. 1.8%; hazard ratio: 1.32 [95% CI: 1.03 to 1.68] P=0.03).
**TRILOGY-ACS[16]**<br><br>**2012**	N=7243 patients with unstable angina or NSTEMI	Randomised controlled trial<br><br>Double-blind	DAPT with prasugrel plus aspirin vs. clopidogrel plus aspirin for up to 30 months	Composite of cardiovascular death, MI or stroke at 17 months	No difference in the primary endpoint with prasugrel vs. clopidogrel (13.9% vs. 16.0%, hazard ratio: 0.91 [95% CI: 0.79 to 1.05] p=0.21).<br><br>Rates of severe and inctracranial bleeding were similar with prasugrel vs. clopidogrel.

**ACCOAST[17]** **2013**	N=4033 patients with NSTEMI	Randomised controlled trial Double-blind	30mg prasugrel pre-angiography followed by 30mg at the time of PCI if PCI indicated vs. placebo pre-angiography followed by 60mg of prasugrel in patient who underwent PCI	Composite of cardiovascular death, MI, stroke, urgent revascularization or glycoprotein IIb/IIIa inhibitor rescue therapy, by day 7 after randomisation	No difference in primary endpoint with prasugrel pre-treatment vs. placebo (hazard ratio: 1.02 [95% CI: 0.84 to 1.25] p=0.81). Increased rate of major bleeds with prasugrel pre-treatment vs. placebo (hazard ratio: 1.90 [95% CI: 1.19 to 3.02] p=0.006).
**ISAR-REACT 5[18]** **2019**	N=4018 patients with STEMI, NSTEMI or unstable angina	Randomised controlled trial Open label	DAPT with prasugrel plus aspirin vs. ticagrelor plus aspirin	Composite of death, MI, or stroke at 1 year	Lower risk of the primary endpoint with ticagrelor vs. prasugrel (9.3% vs. 6.9%; hazard ratio: 1.36 [95% CI: 1.09 to 1.70] P=0.006). No difference in bleeding with ticagrelor vs. prasugrel.
**TWILIGHT[19]** **2019**	N=7119 patients at high bleeding risk 3 months after stent implantation (indication for	Randomised controlled trial Double-blind	After 3 months of DAPT (aspirin plus ticagrelor) randomised to further 9 months of ticagrelor plus	Bleeding Academic Research consortium type 2, 3, or 5 bleeds at 1 year	Lower incidence of primary endpoint after 3 months DAPT vs. 12 months DAPT (4.0% vs.7.1%; hazard ratio: 0.56 [95% CI: 0.45 to 0.68] p<0.001). No difference in the incidence of death, MI, or stroke after 3 months DAPT vs. 12 months DAPT (3.9% vs. 3.9%; hazard ratio: 0.99 [95% CI: 0.78 to 1.25]).

	PCI ACS in 65%)		placebo vs. further 9 months of DAPT (aspirin plus ticagrelor)		
**CADILLAC[20]**	N=2082 patients with ACS	Randomised controlled trial	PCI vs. PCI plus abciximab	Composite of death, reinfarction, disabling stroke, or ischaemia-driven revascularisation of the target vessel at 6 months	The primary endpoint occurred in 20.0% of patient after balloon angioplasty, 16.5% after balloon angioplasty plus abciximab, 11.5% after stenting and 10.2% after stenting plus abxicimab (p<0.001).<br><br>There was no differences between the groups in the rates of death, stroke or reinfarction.<br><br>Differences in the incidence of the primary endpoint were due to differences in the rates of target vessel revascularisation.
**ISAR-REACT 4[21]**<br><br>**2011**	N=1721 patients with acute NSTEMI	Randomised controlled trial<br><br>Double-blind	Abciximab plus unfractionated heparin vs. bivalirudin immediately before PCI	Composite of death, large recurrent MI, urgent target-vessel revascularisation, or major bleeding at 30 days	No difference in the primary endpoint with abciximab vs. bivalirudin (10.9% vs. 11.0%; relative risk: 0.99 [95 CI: 0.74 to 1.32] p=0.94).<br><br>Higher risk of major bleeding with abciximab vs. bivalirudin (4.6% vs. 2.6%; relative risk: 1.84 [95% CI: 1.10 to 3.07] p=0.02).

Abbreviations: ACCOAST = Comparison of Prasugrel at the Time of Percutaneous Coronary Intervention or as Pretreatment at the Time of Diagnosis in Patients with Non-ST Elevation Myocardial Infarction; CADILLAC = Controlled Abciximab and Device Investigation to Lower Late Angioplasty Complications; CLARITY-TIMI = Clopidogrel as Adjunctive Reperfusion Therapy – Thrombolysis in Myocardial Infarction; CURE = Clopidogrel in Unstable Angina to Prevent Recurrent Events; ISAR-REACT = Intracoronary Stenting and Antithrombotic Regimen: Rapid Early Acition for Coronary Treatment; PLATO = Study of Platelet Inhibition and Patient Outcomes; TRILOGY ACS = Targeted Platelet Inhibition to Clarify the Optimal Strategy to Medically Manage Acute Coronary Syndromes; TRITON-TIMI = Trial to Assess Improvement in Therapeutic Outcomes by Optimizing Platelet Inhibition with Prasugrel – Thrombolysis in Myocardial Infarction; TWILIGHT = Ticagrelor With Aspirin or Alone in High-Risk Patients After Coronary Intervention

Table S4: The main randomised studies of antiplatelet treatment strategies after CABG

Trial	Participants	Setting	Treatment	Primary endpoint	Main findings
**POPular CABG[22]** **2020**	N=499 patients who underwent CABG with ≥ 1 SVG (indication for CABG ACS in 31%)	Randomised controlled trial Double-blind	Aspirin plus placebo vs. aspirin plus ticagrelor after CABG	SVG occlusion at 1 year assessed with computerised tomography angiography	No difference in primary endpoint with ticagrelor plus aspirin vs. aspirin 9.6% vs. 10.1%, odds ratio: 0.87 [95% CI: 0.49 to 1.55] p=0.64).
**TARGET[23]** **2020**	N=250 patients who underwent CABG with SVG (indication for CABG ACS in 61%)	Randomised controlled trial Double-blind	Aspirin plus placebo vs. aspirin plus ticagrelor after CABG	SVG occlusion at 1 year assessed with computerised tomography angiography	No difference in primary endpoint with aspirin vs. ticagrelor plus aspirin 17.4% vs. 13.2%, p=0.30).
**DACAB[24]** **2018**	N=500 patients undergoing CABG with SVG (indication for CABG ACS in 66%)	Randomised controlled trial Open label	Aspirin alone vs. aspirin plus ticagrelor vs. ticagrelor alone after CABG	SVG occlusion at 1 year assessed with computerised tomography angiography	Lower risk of primary endpoint ticagrelor plus aspirin vs. aspirin (11.3% vs. 23.5%, p<0.001). No difference in primary endpoint with ticagrelor alone vs. aspirin alone (17.2% vs. 23.5%, p=0.10).

Abbreviations: ACS = acute coronary syndrome; CASCADE = Trial of Clopidogrel After Surgery for Coronary Artery Disease; DACAB = Different Antiplatelet Therapy Strategy After Coronary Artery Bypass Graft Surgery; POPular TAVR = The Effect of Ticagrelor on Saphenous Vein Graft Patency in Patients Undergoing Coronary Artery Bypass Grafting Surgery; SVG = saphenous vein graft; TARGET = Ticagrelor Antiplatelet Therapy to Reduce Graft Events and Thrombosis

Table S5: Long-term antiplatelet therapy for secondary prevention in IHD patients

Trial	Participants	Setting	Treatment	Primary endpoint	Main findings
**CHARISMA** [25]<br><br>**2006**	N = 15,603.<br><br>Mean age 64.9 years.	Randomised clinical trial.<br><br>Aspirin + clopidogrel vs. aspirin alone for primary and secondary prevention in high-risk patients with stable cardiovascular disease.<br><br>Median follow up: 2.3 years.	Clopidogrel 75 mg daily + low-dose aspirin (75 to 162 mg per day) or placebo plus low-dose aspirin.	Composite of cardiovascular death, MI, or stroke.	Among high-risk patients with stable cardiovascular disease, DAPT with aspirin and clopidogrel did not result in a difference in the primary endpoint compared with aspirin monotherapy (6.8% vs 7.3% for placebo plus aspirin group; relative risk 0.93; p = 0.22).<br><br>Moderate bleeding was significantly higher in the clopidogrel group (2.1% vs. 1.3%, p < 0.001).
**HOST-EXAM Extended study** [26]<br><br>**2022**	N = 5,438.<br><br>Mean age 63.5 years.	Randomised clinical trial. Clopidogrel vs. aspirin monotherapy among patients who had completed the required	Clopidogrel 75 mg once daily or aspirin 100 mg once daily.	All-cause mortality, MI, stroke, readmission due to an acute coronary syndrome, and major bleeding.	Clopidogrel monotherapy is superior to aspirin monotherapy as chronic maintenance therapy among patients who have completed the required duration of DAPT therapy after DES PCI.<br><br>Primary end point occurred in 12.8% and 16.9% in the clopidogrel and aspirin groups, respectively (HR 0.74 [95% CI, 0.63–0.86]; P<0.001).

		duration of DAPT post PCI.<br>Median follow-up of 5.8 years.			BARC ≥2 bleeding was less with clopidogrel, 4.5% vs. aspirin, 6.1% (p = 0.016).
**PEGASUS – TIMI 54** [27]<br>**2015**	N = 21,162.<br>Mean age 65 years.	Randomised clinical trial.<br>High and low-dose ticagrelor vs. placebo among patients with prior MI on a background of aspirin therapy.<br>Median follow up: 33 months.	Ticagrelor 90 mg twice daily, ticagrelor 60 mg twice daily, or placebo.	Composite of cardiovascular death, MI, or stroke.	Among aspirin-treated individuals with prior MI, ticagrelor reduced cardiovascular events.<br>The primary endpoint occurred in 7.8% of the ticagrelor 90 mg bid group (HR vs. placebo = 0.85, p = 0.008), 7.8% of the ticagrelor 60 mg bid group (HR vs. placebo = 0.84, p = 0.004), and 9.0% of the placebo group.<br>Ticagrelor was also associated with an increase in TIMI major bleeding (2.6% with ticagrelor 90 mg [HR vs. placebo = 2.7, p < 0.001], 2.3% with ticagrelor 60 mg [HR vs. placebo = 2.3, p < 0.001], and 1.1% with placebo).

Abbreviations: BARC = Bleeding Academic Research Consortium; CI = Confidence Interval; DAPT = Dual Antiplatelet Therapy; DES = Drug Eluting Stent; HR = Hazard Ratio; MI = Myocardial Infarction; PCI = Percutaneous Coronary Intervention; TIMI = Thrombolysis in Myocardial Infarction.

Table S6: Randomised studies of antiplatelet treatment strategies after TAVI

Trial	Participants	Setting	Treatment	Primary endpoint	Main findings
**ARTE[28]** **2017**	N=222 patients undergoing TAVR, without concurrent indication for chronic OAC	Randomised controlled trial<br><br>Open label	Aspirin monotherapy vs. aspirin and clopidogrel after TAVR	Composite of death, MI, stroke, transient ischaemic attack, or major/ life threatening bleed at 3 months follow up	No difference in primary endpoint with DAPT vs. SAPT (15.3% vs. 7.2%, odds ratio: 2.31 [95% CI: 0.95 to 5.62] p=0.065).<br><br>Higher risk of major/ life-threatening bleeds with DAPT vs. SAPT (10.8% vs. 3.6%, odds ratio: 3.22 [95% CI: 1.01 to 10.34] p=0.038) without being associated with lower risk of death (6.3% vs. 3.6%, p=0.37), MI (3.6% vs. 0.9%, p=0.18) or stroke (2.7% vs. 0.9%, p=0.31).
**POPular-TAVI Cohort A[29]** **2020**	N=665 patients undergoing TAVR, without concurrent indication for chronic OAC	Randomised controlled trial<br><br>Open label	Aspirin monotherapy vs. aspirin and clopidogrel for 3 months post-TAVI then aspirin monotherapy thereafter	Two primary endpoints at 12 months follow up:<br>All bleeds (minor, major, life-threatening or disabling).<br><br>Non-procedure related bleeds (including puncture site bleeding)	Lower risk of all bleeds combined with SAPT vs. DAPT (15.1% vs. 26.6%, risk ratio: 0.57 [95% CI: 0.42 to 0.77] p=0.001).<br><br>Lower risk of non-procedure related bleeds with SAPT vs. DAPT (15.1% vs. 24.9%, risk ratio: 0.61 [95% CI: 0.44 to 0.83] p=0.005).<br><br>No difference with SAPT vs. DAPT for cardiovascular deaths, stroke or MI (9.7% vs. 9.9%).

**POPular-TAVI Cohort B**[30]<br><br>**2020**	N=451 patients undergoing TAVR, with pre-existing indication for chronic OAC	Randomised controlled trial<br><br>Open label	OAC alone vs. clopidogrel and OAC for 3 months post-TAVR	Two primary endpoints at 12 months follow up:<br><br>All bleeds (minor, major, life-threatening or disabling).<br><br>Non-procedure related bleeds (including puncture site bleeding)	Lower risk of all bleeds combined with OAC alone vs. OAC and clopidogrel (21.7% vs. 34.6%, risk ratio: 0.63 [95% CI: 0.43 to 0.90] p=0.01.<br><br>Lower risk of non-procedure related bleeds with OAC alone vs. OAC and clopidogrel (21.7% vs. 34.0%, risk ratio: 0.64 [95% CI: 0.44 to 0.92] p=0.02).<br><br>No difference in cardiovascular deaths, MI or ischaemic stroke with OAC alone vs. OAC and clopidogrel (13.4% vs. 17.3%, risk ratio: 0.77 [95% CI for superiority: 0.46 to 1.31]).

Abbreviations: ARTE = Aspirin Versus Aspirin and Clopidogrel Following TAVI; CI = confidence interval; DAPT = dual antiplatelet therapy; MI = myocardial infarction; OAC = oral anticoagulation; POPular-TAVI = Antiplatelet Therapy for Patients Undergoing Transcatheter Aortic Valve Implantation; SAPT = single antiplatelet therapy.

Table S7: Randomised studies of antiplatelet therapy in stroke and TIAs

Trial	Participants	Setting	Treatment	Primary endpoint	Main findings
**CHANCE** [31]<br>**2013**	N = 5,170.<br>Chinese patients at least 40 years of age (mean age 63 years).	Randomised clinical trial.<br>DAPT with aspirin and clopidogrel vs. aspirin alone, administered within 24 hours of minor ischemic stroke or high-risk TIA.<br>Follow up: 90 days.	Clopidogrel 300mg day 1 followed by 75mg daily + aspirin 75mg to 300mg day 1, followed by 75mg daily for the first 21 days.<br>Control: Aspirin only.	Ischemic or haemorrhagic stroke at 90 days.	Short-term (21 days) dual antiplatelet therapy was beneficial after a non-disabling stroke.<br>8.2% of the clopidogrel group vs. 11.7% of the placebo group (HR 0.68; 95% CI, 0.57-0.81; P<0.001).<br>There was no significant difference in bleeding.
**ESPRIT** [32]<br>**2006**	N = 2739.<br>Mean age 63 years.	Randomised clinical trial.<br>DAPT with aspirin and dipyridamole vs aspirin alone in secondary	Aspirin (30-325 mg daily) + dipyridamole 200mg daily combined vs. aspirin alone	Composite of death from all vascular causes, non-fatal stroke, non-fatal MI, or major bleeding.	DAPT with aspirin and dipyridamole was superior to aspirin alone in reducing subsequent ischaemic events after minor stroke or TIA.<br>13% aspirin + dipyridamole vs. 16% aspirin alone (HR 0.80, 95% CI 0.66–0.98).

		prevention of vascular events after ischaemic stroke.<br><br>Treatment administered within 6 months of minor stroke or TIA.<br><br>Mean follow up: 3.5 years.			
**POINT** [33]<br><br>**2018**	N = 4881.<br><br>Enrolled across 269 international sites.<br><br>Mean age 65 years.	Randomised clinical trial.<br><br>DAPT with aspirin and clopidogrel vs. aspirin alone, administered within 24 hours of minor ischemic stroke or	Clopidogrel 75mg daily + aspirin ranged from 50mg to 325mg daily - Median 81mg.<br><br>Control: Aspirin alone.	Primary efficacy outcome: Cardiovascular death, stroke, or MI.<br><br>Primary safety outcome: Major haemorrhage.	Clopidogrel plus aspirin compared with aspirin alone was associated with a reduction in ischemic events, but an increase in major haemorrhage.<br><br>Efficacy outcome: DAPT 5.0% vs. aspirin alone 6.5% (HR 0.75; 95% CI 0.59-0.95; p = 0.02)<br><br>Safety outcome: DAPT 0.9% vs. aspirin alone 0.4% respectively (HR 2.32; 95% CI, 1.10 to 4.87; p = 0.02).

		high-risk TIA.<br>Follow up: 90 days.			
**PRoFess** [34]<br>**2008**	N = 20,332.<br>Mean age 66 years.	Randomised clinical trial.<br>DAPT with aspirin and dipyridamole vs. clopidogrel monotherapy in patients with<br>ischaemic stroke within the last 90 days.<br>Follow up 2.5 years.	Aspirin 25mg + dipyridamole 200mg twice daily vs. clopidogrel 75mg daily	First recurrence of stroke (all cause).	Recurrent stroke rates were similar with both regimes, 9.0% with aspirin + dipyridamole and 8.8% with clopidogrel (HR 1.01; 95% CI 0.92-1.11).<br>Intracranial haemorrhage was significantly more frequent in the aspirin + dipyridamole arm [1.4%], vs. clopidogrel [1.0%] (HR 1.42; 95% CI, 1.11-1.83).
**SPS3** [35]<br>**2012**	N = 3020.<br>Mean age 63 years.	Randomised clinical trial.<br>DAPT with aspirin and clopidogrel vs. aspirin alone in symptomatic	Clopidogrel 75mg + aspirin 325mg daily vs. aspirin only.	Recurrent stroke (including ischemic stroke and intracranial haemorrhage).	DAPT is not superior to aspirin monotherapy for secondary prevention in patients with lacunar/subcortical infarcts: Aspirin 2.7%/patient-year vs. DAPT 2.5%/patient-year (HR 0.92; 95% CI 0.72-1.16, p = 0.15).

		lacunar infarcts on MRI. Treatment commenced within 180 days.<br><br>Mean follow-up: 3.4 years.			DAPT was associated with an increase in bleeding risk: 2.1% vs 1.1% (HR 1.97; 95% CI, 1.41 to 2.71; P<0.001).<br><br>All-cause mortality was higher with DAPT (113) vs aspirin alone (77) (HR 1.52; 95% CI, 1.14 to 2.04; P=0.004)
**SOCRATES** [36]<br><br>**2016**	N= 13,199. Mean age 65.9 years.	Randomised clinical trial.<br><br>Ticagrelor vs. aspirin administered within 24 hours of a low-acuity ischaemic stroke or high-risk TIA.<br><br>Follow up: 90 days.	Ticagrelor 90mg twice daily vs. aspirin 100mg daily.	Death, MI, or stroke.	Ticagrelor monotherapy was not superior to aspirin in reducing MACE in this population.<br><br>Ticagrelor 6.7% vs. aspirin 7.5% (HR 0.89, 95% CI 0.78-1.01, p = 0.07).
**THALES** [37]<br><br>**2020**	N= 11,016.<br><br>Mean age 65 years	Randomised clinical trial.<br><br>DAPT with aspirin and ticagrelor vs.	Ticagrelor (180mg<br><br>loading dose followed by 90mg twice	Time to the occurrence of stroke or death within 30 days.	DAPT with ticagrelor + aspirin reduced subsequent ischemic stroke at 30 days but increased all bleeding.

		aspirin alone within 24hrs of minor ischaemic stroke or high-risk TIA.<br><br>Follow up: 30 days.	daily) + aspirin (300 to 325mg on day 1 followed by 75 to 100mg daily).<br><br>Control: Aspirin + placebo.	Secondary outcomes: Disability and bleeding.	Primary outcome: Aspirin + ticagrelor vs. aspirin, was 5.5% vs. 6.6% (HR 0.83, 95% CI 0.71-0.96, p = 0.02).<br><br>Secondary outcomes for aspirin + ticagrelor vs. aspirin: Severe bleeding: 0.5% vs. 0.1% (p = 0.0001)<br><br>Intracranial haemorrhage or fatal bleeding: 0.4% vs. 0.1% (p = 0.0005)

Abbreviations: CI = Confidence Interval; DAPT = Dual Antiplatelet Therapy; HR = Hazard Ratio; MI = Myocardial Infarction; NSTEMI = Non-ST Elevation Myocardial Infarction; TIA = Transient Ischaemic Attack; TIMI = Thrombolysis in Myocardial Infarction.

Table S8: Randomised studies of antiplatelet therapy in peripheral artery disease

Trial	Participants	Setting	Treatment	Primary endpoint	Main findings
**CAPRIE** [38]<br><br>**1996**	N = 19,185.<br><br>Mean age 62.5 years.	Randomised clinical trial. Clopidogrel vs. aspirin in patients with either recent ischaemic stroke, recent MI, or symptomatic PAD.<br><br>Mean follow-up of 1.91 years.	Clopidogrel 75 mg once daily vs. aspirin 325 mg once daily.	First occurrence of an event in the outcome cluster of ischaemic stroke, MI, or vascular death	Primary endpoint occurred in 5.32% with clopidogrel vs. 5.83% with aspirin (p=0·043).<br><br>There were no major differences in terms of safety.
**COMPASS** [39]<br><br>**2017**	N = 27,395.<br><br>Mean age 68 years.	Randomised clinical trial.<br><br>Low dose rivaroxaban + aspirin in stable atherosclerosis.<br><br>Mean follow up: 23 months.	Rivaroxaban 2.5mg BD + aspirin 100 mg daily, or rivaroxaban 5 mg daily alone, or aspirin 100mg daily alone.	Composite of cardiovascular death, stroke, or MI.	Rivaroxaban + aspirin was associated with fewer adverse cardiovascular events, but more major bleeding events vs. aspirin alone.<br><br>4.1% rivaroxaban + aspirin group vs. 4.9% rivaroxaban alone group vs. 5.4% of the aspirin alone group (p < 0.001 for rivaroxaban + aspirin vs. aspirin alone; p = 0.12 for rivaroxaban alone vs. aspirin alone).<br><br>However, there were more major bleeding events in the rivaroxaban-plus-aspirin group (3.1% vs. 1.9%; HR 1.70; 95% CI, 1.40-2.05; P<0.001)

**EUCLID** [40]<br><br>**2017**	N = 13,885.<br><br>Mean age 67 years	Randomised clinical trial.<br><br>Ticagrelor vs clopidogrel in symptomatic PAD.<br><br>Follow up: 30 months.	Ticagrelor 90 mg twice daily vs. clopidogrel 75mg daily.	Composite of cardiovascular death, MI, or ischemic stroke.	Ticagrelor failed to show superiority to clopidogrel among patients with PAD.<br><br>Primary endpoint occurred in 10.8% of the ticagrelor group vs. 10.6% of the clopidogrel group (p = 0.65).<br><br>Also, no difference in acute limb ischaemia or major bleeding.
**TRA 2 P-TIMI 50** [41]<br><br>**2012**	N = 26,449.<br><br>Mean age 61 years.	Randomised clinical trial.<br><br>Vorapaxar vs. placebo in patients with prior MI (within 2 weeks to 12 months), ischemic stroke (within 2 weeks to 12 months), or PAD (claudication and ankle-brachial index <0.85 or prior revascularisation).<br><br>Follow up: 30 months.	Vorapaxar 2.5 mg daily vs. placebo.	Composite of cardiovascular death, MI, or stroke.	Vorapaxar reduced the risk of cardiovascular death or ischemic events in patients with stable atherosclerosis.<br><br>Primary endpoint occurred in 9.3% in the vorapaxar group vs. 10.5% in the placebo group (HR 0.87; 95% CI, 0.80-0.94; P<0.001).<br><br>Treatment arm resulted in more moderate or severe bleeding, 4.2% vs. 2.5% who received placebo (HR, 1.66; 95% CI, 1.43-1.93; P<0.001).<br><br>There was an increase in intracranial haemorrhage in the vorapaxar group (1.0%, vs. 0.5% in the placebo group; P<0.001).

**VOYAGER PAD** [42]<br><br>**2020**	N = 6,564<br><br>Mean age 67 years.	Randomised clinical trial.<br><br>Rivaroxaban + aspirin vs. placebo + aspirin among patients with lower extremity PAD undergoing revascularisation.<br><br>Follow up: 28 months.	Rivaroxaban 2.5 mg twice daily + aspirin 100mg daily vs. placebo + aspirin.	Cardiovascular death, acute limb ischemia, major amputation, MI, or stroke.	Rivaroxaban + aspirin was superior to aspirin alone at preventing major adverse limb and cardiovascular events (17.3% of the rivaroxaban + aspirin group vs. 19.9% of the placebo + aspirin group (p = 0.0085).<br><br>Similar rates of TIMI major bleeding; however, increased incidence of ISTH major bleeding compared with placebo/aspirin.

Abbreviations: CI = Confidence Interval; DAPT = Dual Antiplatelet Therapy; HR = Hazard Ratio; ISTH = International Society on Thrombosis and Haemostasis; MI = Myocardial Infarction; NSTEMI = Non-ST Elevation Myocardial Infarction; PAD = Peripheral Artery Disease; TIMI = Thrombolysis in Myocardial Infarction.

Table S9: Randomised studies of antiplatelet treatment strategies for COVID patients

Trial	Participants	Setting	Treatment	Primary endpoint	Main findings
**ACTIV -4a** [43]<br>**2020**	N- 562	Randomized, Open Label	Non critically ill Covid patients – Randomised 1:1 to receive PY12 + usual care + no PY12 inhibitor and usual care. For 14 days or until discharge	organ support-free days- the number of index hospitalization days free of cardiovascular or respiratory organ support up to day 21	Ticagrelor was used in 62% and clopidogrel in 38% of participants.<br>The effect of P2Y12 inhibitor on organ support-free days was 0.73 (95% credible interval 0.44 to 1.18)
**REMAP-CAP** ([44]<br>**2021**	N - 1557	Randomized, Open Label	Antiplatelet therapy with either aspirin or a P2Y12 inhibitor, compared with no antiplatelet therapy	Does antiplatelets in critically ill covid- 19 improve organ support–free days (a composite end point of in-hospital mortality and duration of intensive care unit–based respiratory or cardiovascular support) up to day 21	95.7% posterior probability of futility with regard to the odds of improvement in organ support–free days within 21 days
**COVID – PACT** [45]<br>**2020**	N - 672	Randomised, Open label	Full-dose anticoagulation or standard-dose prophylactic anticoagulation. Absent an indication for	composite of death attributable to venous or arterial thrombosis, pulmonary embolism, clinically evident deep venous thrombosis, type 1 myocardial infarction, ischemic stroke, systemic embolic event or acute limb ischemia, or clinically silent deep venous	There were no differences in the primary efficacy or safety end points with clopidogrel versus no antiplatelet therapy.<br>A greater proportion of wins occurred with full-dose anticoagulation (12.3%) versus standard-dose

			antiplatelet therapy, patients were additionally randomly assigned to either clopidogrel or no antiplatelet therapy.	thrombosis, through hospital discharge or 28 days.	prophylactic anticoagulation (6.4%; win ratio, 1.95 [95% CI, 1.08–3.55]; *P*=0.028).
**RECOVERY** [46] **2022**	N- 14 892	Randomised Open label	standard of care plus 150 mg aspirin once per day until discharge or usual standard of care alone	The primary outcome was 28 day mortality	17% patients allocated to aspirin and 17% of 7541 patients allocated to usual care died within 28 days<br><br>composite endpoint of invasive mechanical ventilation or death 21% *vs* 22%;<br><br>Aspirin use resulted in reduction in thrombotic events 4·6% *vs* 5·3% but increase in major bleeding events (1·6% *vs* 1·0%

Abbreviations: ACTIV 4a (Accelerating COVID-19 Therapeutic Interventions and Vaccines-4 Antithrombotics Inpatient Platform Trial) ,REMAP-CAP (Randomized, Embedded, Multifactorial Adaptive Platform Trial for Community-Acquired Pneumonia) RECOVERY – Aspirin in patients admitted to hospital with COVID 19. COVID-PACT (Prevention of Arteriovenous Thrombotic Events in Critically-ill COVID-19 Patients Trial)

REFERENCES

1. Group, A.S.C.; Bowman, L.; Mafham, M.; Wallendszus, K.; Stevens, W.; Buck, G.; Barton, J.; Murphy, K.; Aung, T.; Haynes, R.; et al. Effects of Aspirin for Primary Prevention in Persons with Diabetes Mellitus. *The New England journal of medicine* **2018**, *379*, 1529-1539, doi:10.1056/NEJMoa1804988.
2. McNeil, J.J.; Wolfe, R.; Woods, R.L.; Tonkin, A.M.; Donnan, G.A.; Nelson, M.R.; Reid, C.M.; Lockery, J.E.; Kirpach, B.; Storey, E.; et al. Effect of Aspirin on Cardiovascular Events and Bleeding in the Healthy Elderly. *The New England journal of medicine* **2018**, *379*, 1509-1518, doi:10.1056/NEJMoa1805819.
3. Roshandel, G.; Khoshnia, M.; Poustchi, H.; Hemming, K.; Kamangar, F.; Gharavi, A.; Ostovaneh, M.R.; Nateghi, A.; Majed, M.; Navabakhsh, B.; et al. Effectiveness of polypill for primary and secondary prevention of cardiovascular diseases (PolyIran): a pragmatic, cluster-randomised trial. *Lancet* **2019**, *394*, 672-683, doi:10.1016/S0140-6736(19)31791-X.
4. Steinhubl, S.R.; Berger, P.B.; Mann, J.T., 3rd; Fry, E.T.; DeLago, A.; Wilmer, C.; Topol, E.J.; Observation, C.I.C.f.t.R.o.E.D. Early and sustained dual oral antiplatelet therapy following percutaneous coronary intervention: a randomized controlled trial. *JAMA* **2002**, *288*, 2411-2420, doi:10.1001/jama.288.19.2411.
5. Gwon, H.C.; Hahn, J.Y.; Park, K.W.; Song, Y.B.; Chae, I.H.; Lim, D.S.; Han, K.R.; Choi, J.H.; Choi, S.H.; Kang, H.J.; et al. Six-month versus 12-month dual antiplatelet therapy after implantation of drug-eluting stents: the Efficacy of Xience/Promus Versus Cypher to Reduce Late Loss After Stenting (EXCELLENT) randomized, multicenter study. *Circulation* **2012**, *125*, 505-513, doi:10.1161/CIRCULATIONAHA.111.059022.
6. Colombo, A.; Chieffo, A.; Frasheri, A.; Garbo, R.; Masotti-Centol, M.; Salvatella, N.; Oteo Dominguez, J.F.; Steffanon, L.; Tarantini, G.; Presbitero, P.; et al. Second-generation drug-eluting stent implantation followed by 6- versus 12-month dual antiplatelet therapy: the SECURITY randomized clinical trial. *J Am Coll Cardiol* **2014**, *64*, 2086-2097, doi:10.1016/j.jacc.2014.09.008.
7. Schulz-Schupke, S.; Byrne, R.A.; Ten Berg, J.M.; Neumann, F.J.; Han, Y.; Adriaenssens, T.; Tolg, R.; Seyfarth, M.; Maeng, M.; Zrenner, B.; et al. ISAR-SAFE: a randomized, double-blind, placebo-controlled trial of 6 vs. 12 months of clopidogrel therapy after drug-eluting stenting. *European heart journal* **2015**, *36*, 1252-1263, doi:10.1093/eurheartj/ehu523.
8. Vranckx, P.; Valgimigli, M.; Juni, P.; Hamm, C.; Steg, P.G.; Heg, D.; van Es, G.A.; McFadden, E.P.; Onuma, Y.; van Meijeren, C.; et al. Ticagrelor plus aspirin for 1 month, followed by ticagrelor monotherapy for 23 months vs aspirin plus clopidogrel or ticagrelor for 12 months, followed by aspirin monotherapy for 12 months after implantation of a drug-eluting stent: a multicentre, open-label, randomised superiority trial. *Lancet* **2018**, *392*, 940-949, doi:10.1016/S0140-6736(18)31858-0.
9. Valgimigli, M.; Frigoli, E.; Heg, D.; Tijssen, J.; Juni, P.; Vranckx, P.; Ozaki, Y.; Morice, M.C.; Chevalier, B.; Onuma, Y.; et al. Dual Antiplatelet Therapy after PCI in Patients at High Bleeding Risk. *The New England journal of medicine* **2021**, *385*, 1643-1655, doi:10.1056/NEJMoa2108749.
10. Watanabe, H.; Domei, T.; Morimoto, T.; Natsuaki, M.; Shiomi, H.; Toyota, T.; Ohya, M.; Suwa, S.; Takagi, K.; Nanasato, M.; et al. Effect of 1-Month Dual Antiplatelet Therapy Followed by Clopidogrel vs 12-Month Dual Antiplatelet Therapy on Cardiovascular and Bleeding Events in Patients Receiving PCI: The STOPDAPT-2 Randomized Clinical Trial. *JAMA* **2019**, *321*, 2414-2427, doi:10.1001/jama.2019.8145.
11. Hong, S.J.; Kim, J.S.; Hong, S.J.; Lim, D.S.; Lee, S.Y.; Yun, K.H.; Park, J.K.; Kang, W.C.; Kim, Y.H.; Yoon, H.J.; et al. 1-Month Dual-Antiplatelet Therapy Followed by Aspirin Monotherapy After

Polymer-Free Drug-Coated Stent Implantation: One-Month DAPT Trial. *JACC. Cardiovascular interventions* **2021**, *14*, 1801-1811, doi:10.1016/j.jcin.2021.06.003.
12. Yusuf, S.; Zhao, F.; Mehta, S.R.; Chrolavicius, S.; Tognoni, G.; Fox, K.K.; Clopidogrel in Unstable Angina to Prevent Recurrent Events Trial, I. Effects of clopidogrel in addition to aspirin in patients with acute coronary syndromes without ST-segment elevation. *N Engl J Med* **2001**, *345*, 494-502, doi:10.1056/NEJMoa010746.
13. Sabatine, M.S.; Cannon, C.P.; Gibson, C.M.; Lopez-Sendon, J.L.; Montalescot, G.; Theroux, P.; Claeys, M.J.; Cools, F.; Hill, K.A.; Skene, A.M.; et al. Addition of clopidogrel to aspirin and fibrinolytic therapy for myocardial infarction with ST-segment elevation. *N Engl J Med* **2005**, *352*, 1179-1189, doi:10.1056/NEJMoa050522.
14. Wallentin, L.; Becker, R.C.; Budaj, A.; Cannon, C.P.; Emanuelsson, H.; Held, C.; Horrow, J.; Husted, S.; James, S.; Katus, H.; et al. Ticagrelor versus clopidogrel in patients with acute coronary syndromes. *N Engl J Med* **2009**, *361*, 1045-1057, doi:10.1056/NEJMoa0904327.
15. Wiviott, S.D.; Braunwald, E.; McCabe, C.H.; Montalescot, G.; Ruzyllo, W.; Gottlieb, S.; Neumann, F.J.; Ardissino, D.; De Servi, S.; Murphy, S.A.; et al. Prasugrel versus clopidogrel in patients with acute coronary syndromes. *N Engl J Med* **2007**, *357*, 2001-2015, doi:10.1056/NEJMoa0706482.
16. Roe, M.T.; Armstrong, P.W.; Fox, K.A.; White, H.D.; Prabhakaran, D.; Goodman, S.G.; Cornel, J.H.; Bhatt, D.L.; Clemmensen, P.; Martinez, F.; et al. Prasugrel versus clopidogrel for acute coronary syndromes without revascularization. *N Engl J Med* **2012**, *367*, 1297-1309, doi:10.1056/NEJMoa1205512.
17. Montalescot, G.; Bolognese, L.; Dudek, D.; Goldstein, P.; Hamm, C.; Tanguay, J.F.; ten Berg, J.M.; Miller, D.L.; Costigan, T.M.; Goedicke, J.; et al. Pretreatment with prasugrel in non-ST-segment elevation acute coronary syndromes. *N Engl J Med* **2013**, *369*, 999-1010, doi:10.1056/NEJMoa1308075.
18. Schupke, S.; Neumann, F.J.; Menichelli, M.; Mayer, K.; Bernlochner, I.; Wohrle, J.; Richardt, G.; Liebetrau, C.; Witzenbichler, B.; Antoniucci, D.; et al. Ticagrelor or Prasugrel in Patients with Acute Coronary Syndromes. *The New England journal of medicine* **2019**, *381*, 1524-1534, doi:10.1056/NEJMoa1908973.
19. Mehran, R.; Baber, U.; Sharma, S.K.; Cohen, D.J.; Angiolillo, D.J.; Briguori, C.; Cha, J.Y.; Collier, T.; Dangas, G.; Dudek, D.; et al. Ticagrelor with or without Aspirin in High-Risk Patients after PCI. *N Engl J Med* **2019**, *381*, 2032-2042, doi:10.1056/NEJMoa1908419.
20. Stone, G.W.; Grines, C.L.; Cox, D.A.; Garcia, E.; Tcheng, J.E.; Griffin, J.J.; Guagliumi, G.; Stuckey, T.; Turco, M.; Carroll, J.D.; et al. Comparison of angioplasty with stenting, with or without abciximab, in acute myocardial infarction. *N Engl J Med* **2002**, *346*, 957-966, doi:10.1056/NEJMoa013404.
21. Kastrati, A.; Neumann, F.J.; Schulz, S.; Massberg, S.; Byrne, R.A.; Ferenc, M.; Laugwitz, K.L.; Pache, J.; Ott, I.; Hausleiter, J.; et al. Abciximab and heparin versus bivalirudin for non-ST-elevation myocardial infarction. *The New England journal of medicine* **2011**, *365*, 1980-1989, doi:10.1056/NEJMoa1109596.
22. Willemsen, L.M.; Janssen, P.W.A.; Peper, J.; Soliman-Hamad, M.A.; van Straten, A.H.M.; Klein, P.; Hackeng, C.M.; Sonker, U.; Bekker, M.W.A.; von Birgelen, C.; et al. Effect of Adding Ticagrelor to Standard Aspirin on Saphenous Vein Graft Patency in Patients Undergoing Coronary Artery Bypass Grafting (POPular CABG): A Randomized, Double-Blind, Placebo-Controlled Trial. *Circulation* **2020**, *142*, 1799-1807, doi:10.1161/CIRCULATIONAHA.120.050749.
23. Kulik, A.; Abreu, A.M.; Boronat, V.; Kouchoukos, N.T.; Ruel, M. Ticagrelor versus aspirin and vein graft patency after coronary bypass: A randomized trial. *J Card Surg* **2022**, *37*, 563-570, doi:10.1111/jocs.16189.
24. Zhao, Q.; Zhu, Y.; Xu, Z.; Cheng, Z.; Mei, J.; Chen, X.; Wang, X. Effect of Ticagrelor Plus Aspirin, Ticagrelor Alone, or Aspirin Alone on Saphenous Vein Graft Patency 1 Year After Coronary

Artery Bypass Grafting: A Randomized Clinical Trial. *JAMA* **2018**, *319*, 1677-1686, doi:10.1001/jama.2018.3197.
25. Bhatt, D.L.; Fox, K.A.A.; Hacke, W.; Berger, P.B.; Black, H.R.; Boden, W.E.; Cacoub, P.; Cohen, E.A.; Creager, M.A.; Easton, J.D.; et al. Clopidogrel and Aspirin versus Aspirin Alone for the Prevention of Atherothrombotic Events. *New England Journal of Medicine* **2006**, *354*, 1706-1717, doi:10.1056/NEJMoa060989.
26. Kang, J.; Park, K.W.; Lee, H.; Hwang, D.; Yang, H.-M.; Rha, S.-W.; Bae, J.-W.; Lee, N.H.; Hur, S.-H.; Han, J.-K.; et al. Aspirin Versus Clopidogrel for Long-Term Maintenance Monotherapy After Percutaneous Coronary Intervention: The HOST-EXAM Extended Study. *Circulation* **2023**, *147*, 108-117, doi:doi:10.1161/CIRCULATIONAHA.122.062770.
27. Bonaca, M.P.; Bhatt, D.L.; Cohen, M.; Steg, P.G.; Storey, R.F.; Jensen, E.C.; Magnani, G.; Bansilal, S.; Fish, M.P.; Im, K.; et al. Long-Term Use of Ticagrelor in Patients with Prior Myocardial Infarction. *New England Journal of Medicine* **2015**, *372*, 1791-1800, doi:10.1056/NEJMoa1500857.
28. Rodes-Cabau, J.; Masson, J.B.; Welsh, R.C.; Garcia Del Blanco, B.; Pelletier, M.; Webb, J.G.; Al-Qoofi, F.; Genereux, P.; Maluenda, G.; Thoenes, M.; et al. Aspirin Versus Aspirin Plus Clopidogrel as Antithrombotic Treatment Following Transcatheter Aortic Valve Replacement With a Balloon-Expandable Valve: The ARTE (Aspirin Versus Aspirin + Clopidogrel Following Transcatheter Aortic Valve Implantation) Randomized Clinical Trial. *JACC Cardiovasc Interv* **2017**, *10*, 1357-1365, doi:10.1016/j.jcin.2017.04.014.
29. Brouwer, J.; Nijenhuis, V.J.; Delewi, R.; Hermanides, R.S.; Holvoet, W.; Dubois, C.L.F.; Frambach, P.; De Bruyne, B.; van Houwelingen, G.K.; Van Der Heyden, J.A.S.; et al. Aspirin with or without Clopidogrel after Transcatheter Aortic-Valve Implantation. *N Engl J Med* **2020**, *383*, 1447-1457, doi:10.1056/NEJMoa2017815.
30. Nijenhuis, V.J.; Brouwer, J.; Delewi, R.; Hermanides, R.S.; Holvoet, W.; Dubois, C.L.F.; Frambach, P.; De Bruyne, B.; van Houwelingen, G.K.; Van Der Heyden, J.A.S.; et al. Anticoagulation with or without Clopidogrel after Transcatheter Aortic-Valve Implantation. *N Engl J Med* **2020**, *382*, 1696-1707, doi:10.1056/NEJMoa1915152.
31. Wang, Y.; Wang, Y.; Zhao, X.; Liu, L.; Wang, D.; Wang, C.; Wang, C.; Li, H.; Meng, X.; Cui, L.; et al. Clopidogrel with Aspirin in Acute Minor Stroke or Transient Ischemic Attack. *New England Journal of Medicine* **2013**, *369*, 11-19, doi:10.1056/NEJMoa1215340.
32. Halkes, P.H.; van Gijn, J.; Kappelle, L.J.; Koudstaal, P.J.; Algra, A. Aspirin plus dipyridamole versus aspirin alone after cerebral ischaemia of arterial origin (ESPRIT): randomised controlled trial. *Lancet* **2006**, *367*, 1665-1673, doi:10.1016/s0140-6736(06)68734-5.
33. Johnston, S.C.; Easton, J.D.; Farrant, M.; Barsan, W.; Conwit, R.A.; Elm, J.J.; Kim, A.S.; Lindblad, A.S.; Palesch, Y.Y. Clopidogrel and Aspirin in Acute Ischemic Stroke and High-Risk TIA. *New England Journal of Medicine* **2018**, *379*, 215-225, doi:10.1056/NEJMoa1800410.
34. Sacco, R.L.; Diener, H.C.; Yusuf, S.; Cotton, D.; Ounpuu, S.; Lawton, W.A.; Palesch, Y.; Martin, R.H.; Albers, G.W.; Bath, P.; et al. Aspirin and extended-release dipyridamole versus clopidogrel for recurrent stroke. *The New England journal of medicine* **2008**, *359*, 1238-1251, doi:10.1056/NEJMoa0805002.
35. Investigators, T.S. Effects of Clopidogrel Added to Aspirin in Patients with Recent Lacunar Stroke. *New England Journal of Medicine* **2012**, *367*, 817-825, doi:10.1056/NEJMoa1204133.
36. Johnston, S.C.; Amarenco, P.; Albers, G.W.; Denison, H.; Easton, J.D.; Evans, S.R.; Held, P.; Jonasson, J.; Minematsu, K.; Molina, C.A.; et al. Ticagrelor versus Aspirin in Acute Stroke or Transient Ischemic Attack. *New England Journal of Medicine* **2016**, *375*, 35-43, doi:10.1056/NEJMoa1603060.
37. Johnston, S.C.; Amarenco, P.; Denison, H.; Evans, S.R.; Himmelmann, A.; James, S.; Knutsson, M.; Ladenvall, P.; Molina, C.A.; Wang, Y. Ticagrelor and Aspirin or Aspirin Alone in Acute Ischemic Stroke or TIA. *The New England journal of medicine* **2020**, *383*, 207-217, doi:10.1056/NEJMoa1916870.

38. Committee, C.S. A randomised, blinded, trial of clopidogrel versus aspirin in patients at risk of ischaemic events (CAPRIE). . *Lancet* **1996**, *348*, 1329-1339, doi:10.1016/s0140-6736(96)09457-3.
39. Eikelboom, J.W.; Connolly, S.J.; Bosch, J.; Dagenais, G.R.; Hart, R.G.; Shestakovska, O.; Diaz, R.; Alings, M.; Lonn, E.M.; Anand, S.S.; et al. Rivaroxaban with or without Aspirin in Stable Cardiovascular Disease. *New England Journal of Medicine* **2017**, *377*, 1319-1330, doi:10.1056/NEJMoa1709118.
40. Hiatt, W.R.; Fowkes, F.G.R.; Heizer, G.; Berger, J.S.; Baumgartner, I.; Held, P.; Katona, B.G.; Mahaffey, K.W.; Norgren, L.; Jones, W.S.; et al. Ticagrelor versus Clopidogrel in Symptomatic Peripheral Artery Disease. *New England Journal of Medicine* **2016**, *376*, 32-40, doi:10.1056/NEJMoa1611688.
41. Morrow, D.A.; Braunwald, E.; Bonaca, M.P.; Ameriso, S.F.; Dalby, A.J.; Fish, M.P.; Fox, K.A.A.; Lipka, L.J.; Liu, X.; Nicolau, J.C.; et al. Vorapaxar in the Secondary Prevention of Atherothrombotic Events. *New England Journal of Medicine* **2012**, *366*, 1404-1413, doi:10.1056/NEJMoa1200933.
42. Bonaca, M.P.; Bauersachs, R.M.; Anand, S.S.; Debus, E.S.; Nehler, M.R.; Patel, M.R.; Fanelli, F.; Capell, W.H.; Diao, L.; Jaeger, N.; et al. Rivaroxaban in Peripheral Artery Disease after Revascularization. *New England Journal of Medicine* **2020**, *382*, 1994-2004, doi:10.1056/NEJMoa2000052.
43. Berger, J.S.; Kornblith, L.Z.; Gong, M.N.; Reynolds, H.R.; Cushman, M.; Cheng, Y.; McVerry, B.J.; Kim, K.S.; Lopes, R.D.; Atassi, B.; et al. Effect of P2Y12 Inhibitors on Survival Free of Organ Support Among Non-Critically Ill Hospitalized Patients With COVID-19: A Randomized Clinical Trial. *JAMA : the journal of the American Medical Association* **2022**, *327*, 227-236, doi:10.1001/jama.2021.23605.
44. Bradbury, C.A.; Lawler, P.R.; Stanworth, S.J.; McVerry, B.J.; McQuilten, Z.; Higgins, A.M.; Mouncey, P.R.; Al-Beidh, F.; Rowan, K.M.; Berry, L.R.; et al. Effect of Antiplatelet Therapy on Survival and Organ Support-Free Days in Critically Ill Patients With COVID-19: A Randomized Clinical Trial. *JAMA : the journal of the American Medical Association* **2022**, *327*, 1247-1259, doi:10.1001/jama.2022.2910.
45. Bohula, E.A.; Berg, D.D.; Lopes, M.S.; Connors, J.M.; Babar, I.; Barnett, C.F.; Chaudhry, S.-P.; Chopra, A.; Ginete, W.; Ieong, M.H.; et al. Anticoagulation and Antiplatelet Therapy for Prevention of Venous and Arterial Thrombotic Events in Critically Ill Patients With COVID-19: COVID-PACT. *Circulation* **2022**, *146*, 1344-1356, doi:doi:10.1161/CIRCULATIONAHA.122.061533.
46. Group, R.C. Aspirin in patients admitted to hospital with COVID-19 (RECOVERY): a randomised, controlled, open-label, platform trial. *Lancet* **2022**, *399*, 143-151, doi:10.1016/s0140-6736(21)01825-0.
